# Bimelic Hirayama Disease: Clinical Dilemma Solved by Imaging

**DOI:** 10.1155/2013/606894

**Published:** 2013-03-28

**Authors:** Shalabh Jain, Siddharth Yadav, Swarna Gupta, Ritu Gupta

**Affiliations:** ^1^Department of Radiodiagnosis, VMMC and Safdarjung Hospital, New Delhi 110029, India; ^2^Department of General Surgery, VMMC and Safdarjung Hospital, New Delhi 110029, India; ^3^Department of Otorhinolaryngology, VMMC and Safdarjung Hospital, New Delhi 110029, India

## Abstract

Hirayama disease (juvenile muscular atrophy of distal upper extremity) is a cervical myelopathy predominantly affecting adolescent males. It is characterized by progressive muscular weakness and atrophy of unilateral or asymmetrically bilateral distal upper limbs. We report a case of an 18-year-male painter, who presented with gradually progressive, symmetrical bilateral weakness of hands and forearm for the last two years. On the basis of clinical examination, a provisional diagnosis of lower motor neuron type of symmetrical distal weakness due to heavy metal intoxication was kept. However, imaging studies helped in making a definitive diagnosis of Hirayama disease. The patient was advised cervical collar, and there was no progression in symptoms after six months of followup. Due to the rarity of bilateral symmetrical involvement in Hirayama disease, it remains obscured or unsuspected clinically, and MRI plays a pivotal role in diagnosis.

## 1. Introduction

Hirayama disease, also termed juvenile muscular atrophy of the unilateral upper extremity, is a type of cervical myelopathy related to flexional movements of the neck [1–6]. Although the underlying causative mechanism remains unclear, it is postulated that the myelopathy occurs due to forward displacement of the posterior cervical dural sac when the neck flexes, leading to the compression of the cervical cord [1–6]. Characteristically, the affected patients are 15–25-year-old males with insidious onset, gradually progressive unilateral muscular weakness and atrophy affecting hands and forearm, with sparing of brachioradialis muscle giving an appearance of oblique amyotrophy [7]. The amyotrophy is unilateral in most patients or asymmetrically bilateral in some. Only a few cases of Hirayama disease with bilateral symmetric involvement have been reported in the literature, thus highlighting the significance of imaging in such patients. We report a case of bilateral symmetrical pure motor atrophy diagnosed to be Hirayama disease on flexion cervical spine magnetic resonance imaging. 

## 2. Case History

An 18-year-old-male, right-handed painter (exposure to lead) presented with gradually progressive, symmetrical bilateral upper limb weakness for the last two years. It started as inability to grip heavy objects and progressed to inability to hold food and button/unbutton his shirt over a period of one year. However, there was no difficulty in lifting his arm above the shoulder. There was no history of sensory loss. Weakness was confined to upper limbs with normal bladder and bowel functions. There was no history of any neck pain, trauma, preceding fever, and rashes or any hypoaesthetic patches. There was no other feature suggestive of lead intoxication like recurrent pain abdomen, vomiting, constipation, or behavioural changes. Patient had a normal development history with normal school performance. Patient was nondiabetic and did not have other comorbidity. His coworkers and family members did not have similar complaints.

**Figure 1:**
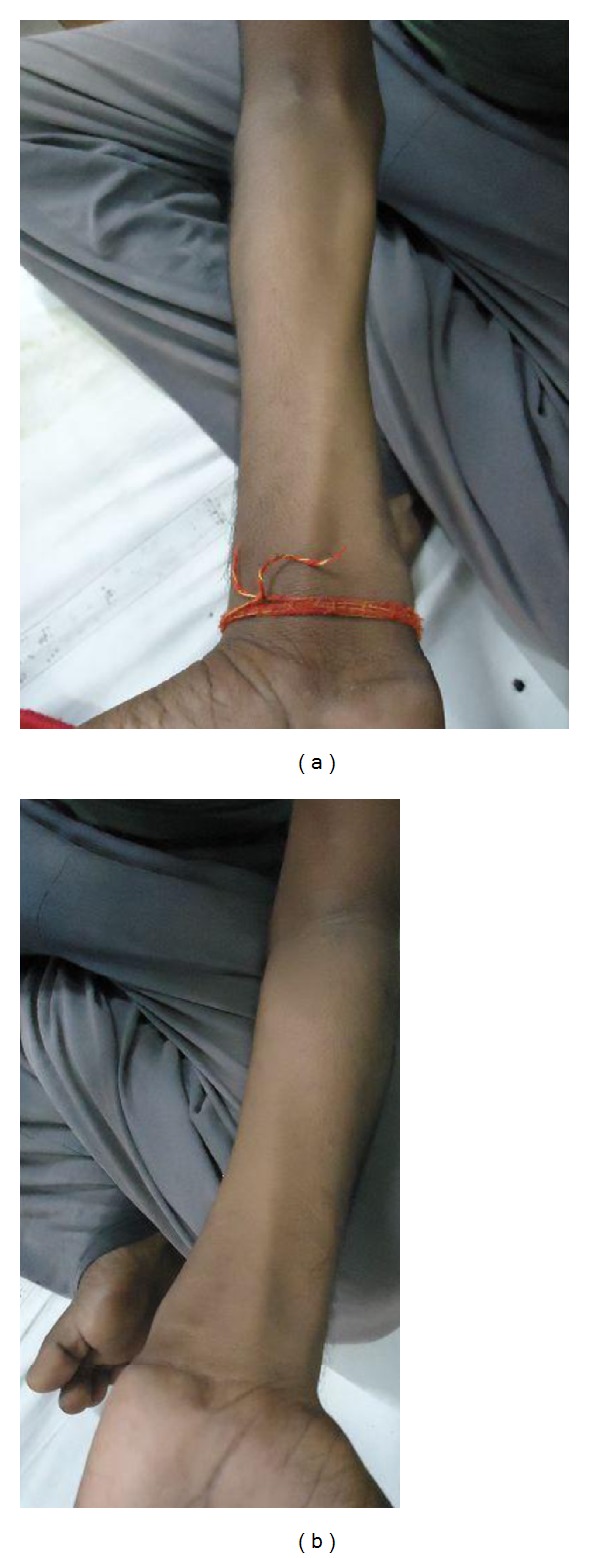
Symmetrical bilateral atrophy of forearm muscles with sparing of Brachioradialis.

**Figure 2:**
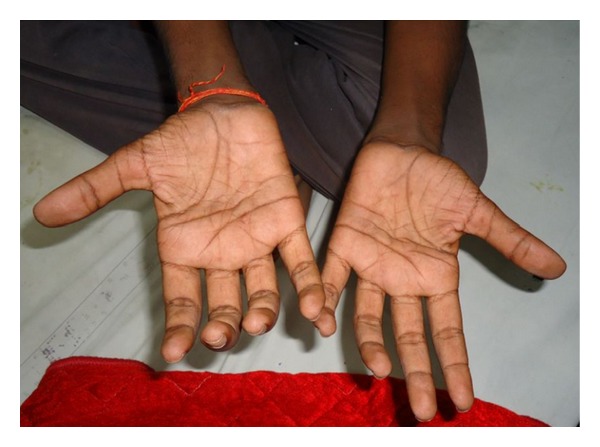
Symmetrical bilateral atrophy of hand muscles.

On examination, there was striking muscular atrophy affecting bilateral hands and forearm except the brachioradialis (Figures 1 and 2). The bulk of arms and shoulders was maintained. Supinator reflex was equivocal bilaterally but both biceps and triceps reflex were preserved as was the sensory system of both upper limbs. Examination of bilateral lower limb, higher mental function, cerebellar function, cranial nerves, and spine was normal. There was no finding suggestive of raised intracranial tension. The rest of the systemic examination was unremarkable.

On the basis of history and clinical examination, a lower motor neuron type of gradually progressive distal weakness and atrophy localized to distal upper extremity was considered (motor polyneuropathy) most probably due to heavy metal intoxication (lead).

## 3. Investigations

Haemoglobin level, erythrocyte sedimentation rate, total and differential leukocyte count, liver function tests, renal function tests, blood sugar, and electrolytes of patient were within normal limits. Peripheral smear showed normocytic normochromic picture with no basophilic stippling. Muscle enzymes level like creatinine phosphokinase and lactate dehydrogenase was normal. Skiagram cervical spine did not reveal any abnormality. 

**Figure 3:**
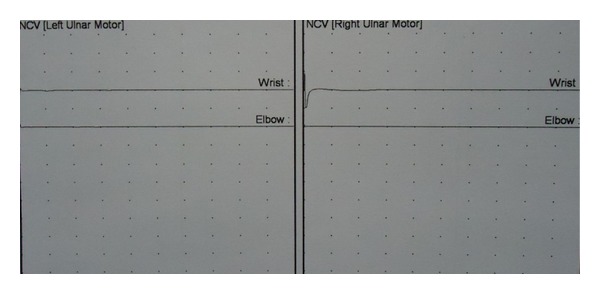
Nerve conduction study showing no motor response in bilateral ulnar nerves.

Nerve conduction velocity (NCV) of motor nerves showed no response in bilateral median and ulnar nerves. F waves were absent. Sensory conduction velocities of ulnar, median and examination of other nerves of upper and lower limbs were normal. These findings were suggestive of bilateral ulnar and median nerve pure motor neuropathy (Figure 3).

Since our patient was a painter by occupation, serum lead and arsenic levels analysis were done, both of which were within normal limits. Urine analysis to detect heavy metal toxins was also normal forcing us for further investigations. Serum vitamin B12 level, folate levels and thyroid function tests were normal. Serologies for antinuclear antibody, rheumatoid factor, human immunodeficiency virus (HIV), and hepatitis viruses B and C were negative. Cerebrospinal fluid analysis was normal.

**Figure 4:**
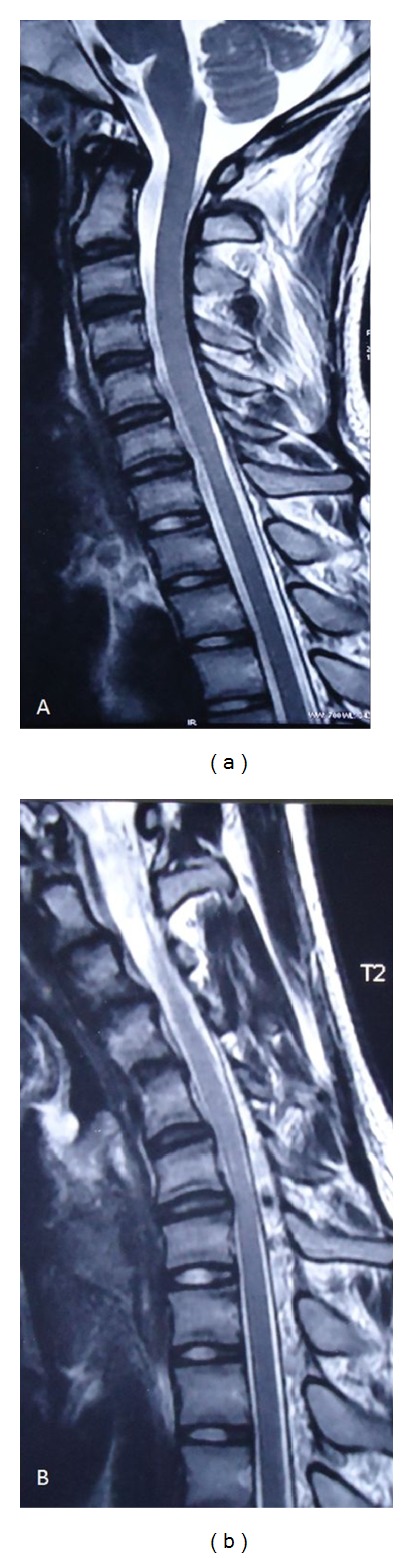
(a) Neutral position MRI of cervical spine T2W image reveals mild focal atrophy of cervical cord at C6-C7 levels with loss of attachment between the posterior dural sac and subjacent lamina. (b) Flexion MRI of cervical spine T2W image reveals characteristic anterior displacement of the posterior wall of cervical dural canal reaching up to spinal cord. Prominent posterior epidural spaces with multiple flow voids suggestive of prominent venous plexus are present.

Magnetic resonance imaging (MRI) (Figure 4) of cervical spine was done. In neutral position, MRI revealed mild symmetrical atrophy of lower cervical spinal cord at C6-C7 levels with loss of attachment between the posterior dural sac and subjacent lamina. No altered intramedullary signals were noted. Intervertebral discs showed mild dessication changes without any disc bulge. On the basis of above findings, suspicion of Hirayama disease was kept and MRI in flexion position was done confirming the diagnosis. In flexion, there was a characteristic anterior displacement of the posterior wall of cervical dural canal reaching up to spinal cord. Prominent posterior epidural space was seen with multiple flow voids suggestive of prominent venous plexus. 

On the basis of clinical and radiological findings, the diagnosis of bimelic hirayama disease was made and the patient was kept conservatively on cervical collar and physiotherapy. On 6 months of follow up, the disease has not shown any progression.

## 4. Discussion

Hirayama disease is a benign disorder and has an initial progressive course of one to three years followed by a stationary stage. It affects young males between the age of 15 and 25 years. The clinical features include insidious onset, predominantly unilateral upper extremity weakness and atrophy, and cold paresis without sensory or pyramidal tract involvement [1–5]. This was first reported by Hirayama et al. [2] in 1959 and bears his name. It is believed that the primary pathogenetic mechanism is forward displacement of the posterior wall of the lower cervical dural canal in neck flexion [6, 8]. Normally the difference in length of dural canal between extension and flexion from T-1 to the top of the atlas is 1.5 cm at the anterior wall and 5 cm at the posterior wall [9]. The slack of the dura can compensate for the increased length in flexion in normal adults. But in Hirayama disease, the dural canal is no longer slack because of an imbalance in growth of the vertebrae and the dura mater. Therefore, a tight dural canal is formed, which cannot compensate for the increased length of the posterior wall during flexion. This causes an anterior shifting of the posterior dural wall, with consequent compression of the cord. This compression may cause microcirculatory disturbances in the territory of the anterior spinal artery or in the anterior portion of the spinal cord. The chronic circulatory disturbance resulting from repeated or sustained flexion of the neck may produce necrosis of the anterior horns, which are most vulnerable to ischemia [1]. Since only anterior horns are affected, there is primarily motor involvement.

The disease is unilateral in most patients, asymmetrically bilateral in some, and is rarely symmetric [10]. Only few cases of Hirayama disease with bilateral symmetric involvement have been reported in the literature [10]. When bilateral, a very strong clinical suspicion is required to diagnose Hirayama disease. Syringomyelia, spinal cord tumors, poliomyelitis, multifocal motor neuropathy, and toxic neuropathies should be excluded in such cases. Our case was initially diagnosed as a case of toxic neuropathy as he was a painter by occupation, which could have exposed him to lead. But his serum lead levels were normal. MRI in neutral position revealed mild atrophy of lower cervical cord; however, no signal intensity changes were noted in cord. Due to the high index of suspicion of Hirayama disease, MRI of cervical spine was done in flexion position which revealed the correct and final diagnosis.

Though Hirayama disease is self-limiting, early diagnosis is still necessary because placement of a cervical collar will prevent neck flexion, which has been shown to stop disease progression [11]. 

## 5. Conclusion

Bimelic symmetrical involvement in Hirayama disease is a very rare occurrence with only few cases reported and may remain undiagnosed due to a common understanding that Hirayama disease has unilateral or asymmetric bilateral presentation. So, a high index of suspicion should be kept clinically as well as while performing neuroimaging studies, which may prompt us to perform dynamic imaging studies. Early detection of disease is of paramount important as limitation of flexion movements using cervical collar helps in preventing the progression of disease.
